# Ethnic Differences in the Prevalence of High Homocysteine Levels Among Low-Income Rural Kazakh and Uyghur Adults in Far Western China and Its Implications for Preventive Public Health

**DOI:** 10.3390/ijerph120505373

**Published:** 2015-05-19

**Authors:** Shuxia Guo, Hongrui Pang, Heng Guo, Mei Zhang, Jia He, Yizhong Yan, Qiang Niu, Muratbek, Dongsheng Rui, Shugang Li, Rulin Ma, Jingyu Zhang, Jiaming Liu, Yusong Ding

**Affiliations:** Key Laboratory of Xinjiang Endemic and Ethnic Diseases, School of Medicine, Shihezi University, Beier Road, Shihezi City, Xinjiang Uyghur Autonomous Region 832000, China; E-Mails: 15909938133@163.com (H.P.); guoheng@shzu.edu.cn (H.G.); zmberry@foxmail.com (M.Z.); hejia123.shihezi@163.com (J.H.); erniu19880215@sina.com (Y.Y.); niuqiang1214@163.com (Q.N.); murat08123@163.com (Mu.); ruidongsheng@gmail.com (D.R.); lishugang@ymail.com (S.L.); marulin@126.com (R.M.); yfyxxzjy@126.com (J.Z.); liujiaming@shzu.edu.cn (J.L.); 13399931625@163.com (Y.D.)

**Keywords:** Kazakh, Uyghur, serum homocysteine, hypertension, prevalence

## Abstract

*Objective*: Homocysteine (Hcy) is a relevant biomarker of vascular disease: serum Hcy concentrations will increase the risk of systolic hypertension, whereas hyperhomocysteinemia (HHcy) has a synergistic effect with hypertension and increases the risk of cardiovascular disease. However, information has primarily been gathered from high-income and urban settings, and little is known regarding low-income rural settings. This study focused on a low-income rural and nomadic minority residing in far western China. Hcy levels were tested, and the prevalences of HHcy and H-type hypertension were investigated in this population. *Methods*: This study used a stratified cluster random sampling method, selecting 2,180 individuals as subjects from Kazakh and Uyghur inhabitants (≥25 years old) of 18 villages in Xinjiang, China, which is approximately 4407 km from the capital, Beijing. Hcy levels were determined using a double reagent enzymatic cycling method. HHcy (Hcy > 10 μmol/L) was defined by the criteria of the American Heart Association. *Results*: The Kazakh geometrical mean of Hcy was 13.34 μmol/L, and the Uyghur mean was 13.75 μmol/L; the mean values were higher in males than in females of both ethnicities (15.99 μmol/L *vs.* 11.63 μmol/L; 15.71 μmol/L *vs.* 11.91 μmol/L, respectively, *p* < 0.01). The serum levels of Hcy increased with increasing age in both ethnicities, and except for Kazakh individuals >65 years old, Hcy serum levels were higher in males than in females in all age groups of both ethnicities, with a *p* value less than 0.01. The Kazakh prevalence of HHcy was 80.0%, and the Uyghur prevalence was 78.2%; the male prevalence was higher than that in females for both ethnicities (93.5% *vs.* 69.6%; 90.8% *vs.* 64.6%, respectively, *p* < 0.05). Among the Kazakh, the prevalence of hypertension was 35.1%, and the prevalence was higher in males than in females (44.3% *vs.* 28.1%, *p* < 0.001); 87.6% of the Kazakh individuals had H-type hypertension, and the prevalence was higher in males than in females (95.0% *vs.* 80.0%, *p* < 0.05). In Uyghur, the prevalence of hypertension was 30.6%, and the prevalence was higher in males than in females (37.9% *vs.* 22.8%, *p* < 0.001); 88.0% of the Uyghur individuals had H-type hypertension, and the prevalence was higher in males than in females (93.9% *vs.* 79.1%, *p* < 0.05). *Conclusions*: HHcy was found to be common among the Kazakh and Uyghur. The prevalences of HHcy and H-type hypertension were high among both ethnicities and differed depending on gender and age. Community interventions should be conducted to improve public health conditions among the Kazakh and Uyghur in Xinjiang.

## 1. Introduction

Homocysteine (Hcy) is an intermediate product of methionine metabolism and is generated by the demethylation of methionine, a sulfur-containing amino acid also called 2-methyl-4-mercaptobutyric acid. Under normal conditions, Hcy in the human body is maintained in a strict homeostasis between generation and clearance, with a fasting plasma or serum Hcy concentration in adults of approximately 5–15 μmol/L [1]. When Hcy levels exceed the normal upper limit because of hereditary or acquired factors, this is referred to as hyperhomocysteinemia (HHcy). However, Hcy levels differ by ethnicity due to different regions and gene expression patterns, and the reported ranges from different nations and regions vary. For example, Hcy levels as high as > 15 μmol/L have been described by scholars from Europe and Iran prior to 2007 [2,3]. However, in 2006, levels > 10 μmol/L in ischemic stroke and transient ischemic attacks in patients with stroke prevention guidelines were released by the American Society of Hypertension (AHA) and the American Stroke Association (ASA) [4]; in this guide, HHcy is listed as one of the major risk factors for stroke.

The incidence of HHcy is influenced by a variety of factors, including genetic factors, nutritional factors (B vitamin deficiency and folic acid deficiency), lifestyle behaviors (smoking and drinking), drugs, disease, lower estrogen levels [5] and even environmental factors, such as high levels of lead in the blood [6,7]. In recent years, based on numerous studies, HHcy has been considered to be an independent risk factor for atherosclerosis and cardiovascular disease [8,9,10]. When Hcy levels are elevated by 5 μmol/L, the risk of cerebrovascular disease increases by 59%, and the risk of coronary heart disease increases by 32% [11]. However, when Hcy levels are reduced by 3 μmol/L, the risk of stroke decreases by 24%, and the risk of ischemic heart disease decreases by 16% [11]. In contrast, Wang *et al*. reported no significant association between Hcy and the presence of coronary heart disease (CHD) after adjusting for covariates in Chinese hypertensive patients [12]. Currently in China, research on Hcy is mainly focused on the “correlation with disease”, and most studies are conducted in Beijing, Shanghai, Tianjin and the eastern parts of the more developed regions. Conversely, there have been few investigations on the epidemiology and metabolism of Hcy, and those conducted in remote northwestern China and in ethnic minorities are scarce [13].

China is a multi-ethnic country, and 13 main ethnic groups have been reported in the Xinjiang Uyghur Autonomous Region, of which the Kazakh and Uyghur are two large inhabitant minority groups. Because of the limited resources available for public health and poor transportation, there has been a lack of serious investigation analyzing local public health needs, including the prevalence of HHcy and related diseases such as hypertension and cardiovascular disease. Nonetheless, because of differences in religion, culture, lifestyle, diet, and genetic background between these ethnic groups, characterizing the prevalence of HHcy may reveal valuable information for making appropriate policies in preventive public health for the inhabitants of Xinjiang.

Accordingly, the prevalence of HHcy among Kazakh and Uyghur populations has not yet been addressed. Therefore, this study aimed to investigate this prevalence based on data from two rural and nomadic minorities residing in far western China to provide proper guidelines for a cardiovascular and cerebrovascular disease prevention program.

## 2. Methods

### 2.1. Ethics Statement

The Institutional Ethics Review Board (IERB) at the First Affiliated Hospital of Shihezi University School of Medicine approved the study (IERB No. SHZ2010LL01). Standard university hospital guidelines including informed consent, voluntary participation, confidentiality, and anonymity were followed. All the participants provided written informed consent before the study began.

### 2.2. Settings and Study Population

The survey was conducted from 2011 to 2012 in Yili (Kazakh) and Kashi (Uyghur) prefectures, respectively, which are approximately 4407 km (2739 miles) from Beijing. Approximately 98% of the population in these prefectures is of the Muslim Kazakh or Uyghur minorities. A multistage (prefecture-county-township-village) stratified cluster random sampling method was used to select participants. At the beginning of the study, we chose these two representative prefectures (Yili and Kashi) according to the geographical distribution of the minority populations in Xinjiang, a province in northwest China. We randomly selected one county in each prefecture and one township from each county (Nalati Township in Xinyuan County and Jiangbazi Township in Jiashi County). At the last stage, a stratified sampling method was used to select corresponding villages in each township (six villages in Nalati Township and 12 villages in Jiangbazi Township). We interviewed local Kazakh and Uyghur inhabitants aged 25 years or older who had resided in the village for at least 6 months and successfully interviewed a total of 7211 individuals (3390 Kazakh and 3821 Uyghur). The overall response rate was 91.3% (89.7% for Kazakh and 92.5% for Uyghur). Due to the limited funding available for this study, to meet the required sample size, we adopted a stratification sampling method according to sex and age through proportional random sampling and random number tables. In particular, we selected a total of 1003 individuals (29.6%) and 1177 individuals (30.8%) from the Kazakh and Uyghur populations, respectively, as the subjects for this study.

### 2.3. Questionnaire Survey

A self-developed questionnaire was applied to collect detailed information from all the respondents during a face-to-face interview. The questionnaire consisted of the demographic information of the respondents (such as age, gender, ethnicity, education level, and marital status) and personal lifestyle (such as smoking, alcohol intake, physical activity, and dietary habits).

### 2.4. Physical Examination and Detection of Serum Hcy

A physical examination, including measurements of height, weight, waist circumference, hip circumference, and systolic and diastolic blood pressure, was performed. A total of 4–5 mL of fasting venous blood was collected from the participants. Within 1 h, the samples were centrifuged at 3000 r/min for 10 min to separate the serum, which was stored at −80 °C. Hcy was detected by the double loop method using a DXC-800 automatic biochemical analyzer (Beckman, pasadena, Cal., USA) with a serum homocysteine precision of 0.1 μmol/L. The kit used was supplied by Hangzhou Franc Medical Devices Co. (Hangzhou, China)

### 2.5. Definitions

(1) In 2006, the American Heart Association and the Joint Commission Stroke (AHA and ASAC) jointly launched the “About ischemic stroke and transient ischemic attack (TIA) Prevention Guide”. In this guide, a standard baseline serum Hcy level of > 10 μmol/L was used as the HHcy diagnostic criterion [4]. (2) The diagnostic criteria of hypertension were obtained from the 2010 “Chinese Hypertension Prevention Guide” and the World Health Organization and the International Society of Hypertension (WHO/ISH) “hypertension guidelines.” With these diagnostic criteria, the mean of three measurements of sitting blood pressure was evaluated based on the following standards: systolic blood pressure ≥ 140 mmHg or diastolic blood pressure ≥ 90 mmHg and patients who were diagnosed with essential hypertension or who had used oral antihypertensive drugs within two weeks [14]. When investigating abnormal blood pressure, all previous original hypertensive patients with idiopathic statistics were considered. H-type hypertension was defined as a hypertension patient with concomitant serum homocysteine ≥ 10 μmol/L [15].

### 2.6. Statistical Analysis

A databank was created using EpiData software (EpiData Association, Odense, Denmark, http://www.epidata.dk/). The data were analyzed using SPSS (Statistical Program for Social Sciences, version 17.0, (SPSS Inc., Chicago, IL, USA, 2008). Continuous variables are presented as the means ± standard deviations (M ± SD) and were analyzed using a *t*-test. The serum Hcy levels displayed a positively skewed distribution. After logarithmic (lg10) transformation, the data were approximately normally distributed with a geometric mean (G) and corresponding 95% confidence intervals (95% CI) on behalf of the serum Hcy average. Categorical variables were expressed as numbers or percentages and were analyzed using the Chi-square test and trend test. All statistical tests were two-sided, and differences were considered statistically significant at *p* values < 0.05.

## 3. Results

### 3.1. Basic Characteristics of the Study Populations

Among the 1003 Kazakh adults, there were 433 men (43.2%) and 570 women (56.8%), with an average age of 42.18 ± 11.39 years. Among the 1177 Uyghur adults, there were 610 men (51.8%) and 567 women (48.2%), with an average age of 48.07 ± 15.18 years. The values of average age, prevalence of hypertension, Hcy, systolic blood pressure (SBP), diastolic blood pressure (DBP) and gender differed significantly among the ethnicities (*p* < 0.001 for each comparison), with the Kazakh adults having higher SBP, DBP and prevalence of hypertension compared to the corresponding Uyghur adults (Table 1).

### 3.2. Prevalence of HHcy

Table 2 shows the prevalence of HHcy in these two ethnic groups based on the American Heart Association criteria stratified by gender and age. In both the Kazakh and Uyghur populations, the prevalence of HHcy was higher in males than in females (*χ^2^*_Uyghur_ = 118.814, *p* < 0.001; *χ^2^*_Kazakh_ = 87.603, *p* < 0.001). With increasing age, the prevalence of HHcy also increased in males, females, and the total populations of both ethnicities. Except for the 45–54 and >65 year age groups of Kazakh, the prevalences of HHcy in the males of both ethnicities were significantly higher than those of the corresponding females among all age groups (*p* < 0.05).

### 3.3. Ethnic Differences in the Prevalence of HHcy between Kazakh and Uyghur Adults

Table 2 shows that the prevalence rates of HHcy in the Uyghur men, women and the general population were 90.8%, 64.6% and 78.2%, respectively; these rates were 93.5%, 69.6% and 80.0%, respectively, in the Kazakh. The prevalence rates of HHcy in both male and female Kazakhs were significantly greater than those of the corresponding male and female Uyghurs in the >35-year age group (*p* < 0.05 for both comparisons). The prevalence rates of Hhcy in female Kazakhs were significantly greater than those of the female Uyghurs in the >55-year age group (*p* < 0.05). However, in the other age groups, the prevalence rates of HHcy were identical in both the male and female Kazakh and Uyghur adults. Table 3 displays the prevalence of H-type hypertension among the 1003 Kazakh.

**Table 1:** Baseline characteristics of the study population.

Characteristic	Kazakh			Uyghur			Total		
	Male	Female	Total	Male	Female	Total	Male	Female	Total
n	433	570	1003	610	567	1177	1043	1137	2180
Age (years) *	43.47	41.20	42.18	49.56	46.47	48.07	47.03	43.83	45.36
	(12.26)	(10.58)	(11.39)	(15.47)	(14.72)	(15.18)	(14.53)	(13.08)	(13.88)
Hcy (μmol /L) ^&^	15.99	11.63	13.34	15.71	11.91	13.75	15.83	11.77	13.56
	(10.92~23.42)	(8.57~15.76)	(9.17~19.40)	(10.24~24.11)	(7.92~17.93)	(8.85~21.37)	(10.51~23.83)	(8.21~16.87)	(8.89~20.47)
SBP (mmHg) *	136.47	128.93	132.18	130.84	122.60	126.87	133.18	125.77	129.31
	(23.60)	(20.99)	(22.46)	(19.77)	(21.64)	(21.09)	(21.61)	(21.54)	(21.88)
DBP (mmHg) *	86.34	82.76	84.30	80.27	75.72	78.08	82.79	79.25	80.94
	(14.11)	(13.32)	(13.77)	(12.69)	(13.50)	(13.28)	(13.63)	(13.86)	(13.86)
Hypertension **	202	200	402	246	163	409	448	363	811
	(46.65)	(35.09)	(40.08)	(40.33)	(28.75)	(34.75)	(42.95)	(31.93)	(37.20)

Notes: SD means standard deviation. GM represents geometric mean, CI depicts confidence interval * =Data are expressed as means (SD). ** =Data are expressed as n (%). ^&^ =Data are expressed as GM (95% CI).

**Table 2:** Prevalence of hyperhomocysteinemia among Kazakh and Uyghur adults stratified by age and gender.

Age (years)	American Heart Association (Hcy ≥ 10 μmol/L)					
	Kazakh			Uyghur		
	Male	Female	Total	Male	Female	Total
25~34	122	113	235	123	77	200
	(89.7) *	(57.4)	(70.6)	(89.8) *	(54.2)	(71.7)
35~44	117	124	241	118	91	209
	(94.4) ^&,^*	(68.1) ^&^	(78.8)	(86.8) *	(52.0)	(67.2)
45~54	87	108	195	102	62	164
	(94.6) *	(78.3)	(84.8)	(89.5) *	(68.1)	(80.0)
55~64	52	35	87	97	67	164
	(96.3)	(97.2) ^&^	(96.7)	(93.3) *	(83.8)	(89.1)
>65	27	17	44	114	69	183
	(100.0)	(100.0)	(100.0)	(95.8) *	(87.3)	(92.4)
Overall	405	397	802	554	366	920
	(93.5) *	(69.6)	(80.0)	(90.8) *	(64.6)	(78.2)
*χ^2^* trend	6.143	39.468	48.640	7.393	49.992	65.718
*p* value	0.189	<0.001	<0.001	0.117	<0.001	<0.001

Note: Data are expressed as n (%). Descriptive characteristics were compared by the *χ^2^* test and the trend test. * = *p* < 0.05 *versus* females from the same age group and ethnicity. ^&^ = *p* < 0.05 *versus* different ethnic groups of the same sex and age.

**Table 3:** Prevalence of H-type hypertension in Kazakh and Uyghur rural adults according to gender.

Sex	Kazakh (Survey)			Kazakh (Hypertensive Patients)			Uyghur (Survey)			Uyghur (Hypertensive Patients)		
	n	Patients	Prevalence	n	Patients	Prevalence	n	Patients	Prevalence	n	Patients	Prevalence
Female	570	160	28.1	200	160	80.0	567	129	22.8	163	129	79.1
Male	433	192	44.3 *	202	192	95.0 ^#^	610	231	37.9 *	246	231	93.9 ^#^
Total	1003	352	35.1	404	352	87.6	1177	360	30.6	409	360	88.0

Note: ^#^ = Data are expressed as n (%). Descriptive characteristics were compared by the *χ^2^* test and the trend test. * = *p* < 0.05 versus females from the same ethnic group.

The average hypertension prevalence was 35.1% (352/1003), and the prevalence rate was higher in males than in females: 44.3% *vs.* 28.1% (*χ^2^* = 28.602, *p* < 0.001). In total, 87.6% of the hypertensive patients had H-type hypertension, and the prevalence was higher in males than in females: 95.0% *vs.* 80.0% (*χ^2^* = 20.900, *p* < 0.001). In the Uyghur, the average hypertension prevalence was 30.6% (360/1177), and the prevalence was higher in males than in females: 37.9% *vs.* 22.8% (*χ^2^* = 31.632, *p* < 0.001). In total, 88.0% of the hypertensive patients had H-type hypertension, and the prevalence was higher in males than in females: 93.9% *vs.* 79.1% (*χ^2^* = 20.258, *p* < 0.001).

## 4. Discussion and Conclusions

Hcy is a risk factor for many diseases, such as hypertension, diabetes, coronary heart disease, Alzheimer’s disease, kidney disease, ischemic cerebrovascular disease and cerebral hemorrhage [5]. Peripheral vascular disease is closely related to a variety of chronic diseases and pregnancy-induced hypertension syndrome. With the rapid development of the global economy, the living standards of various populations have greatly improved. Similarly, cardiovascular disease (including coronary heart disease, atherosclerotic heart disease, and stroke) has demonstrated an increasing trend in incidence, has become a serious threat to human health, is one of the most common diseases affecting the quality of life and is the leading cause of death worldwide [16]. As the leading cause of death in China, cardiovascular disease has significant associated medical costs [17]. Sun *et al.* have reported that elevated plasma Hcy concentrations increase the risk of systolic hypertension [18], whereas HHcy has a synergistic effect with hypertension and can lead to an increased risk of cardiovascular disease. According to reports, H-type hypertension accounts for approximately 75% of cases of adult hypertension in China, translating into approximately 150 million H-type hypertensive patients [5]. Compared to Western populations, the levels of Hcy are significantly higher in the Chinese population [19], which has a high population base suffering from hypertension [15], and the distribution is similar to that of high blood pressure. Hcy is also more common in southern populations than in northern populations in China [20,21,22,23], but the incidence is higher in rural areas than in urban areas [24]; differences in dietary habits between rural and urban areas may lead to observed differences in homocysteine. Our earlier epidemiological survey showed that the prevalence of hypertension reached 36.7% in Xinjiang Kazakhs and 27.8% in Uyghurs [25]. Therefore, epidemiological studies highlighting the burden of HHcy in the Kazakh and Uyghur populations are important for the prevention and treatment of hypertension.

The current study intended to investigate the epidemiological features of HHcy in rural Kazakh and Uyghur adults in northwest China. Our study showed HHcy prevalences of 80.0% and 78.2% for the Uyghur and Kazakh populations, respectively, which were higher than the prevalences observed in other regions in China and significantly higher than the prevalence in the Han population (45.0%) [20]. In the Kazakh and Uyghur populations, the prevalence of HHcy increased with age and showed a gradual increasing trend. This trend may be associated with increased age, decreased cystathionine enzyme activity, decreased Hcy metabolism (which decreases renal clearance), low vitamin B levels and reduced folic acid bioavailability [26,27,28]. The HHcy age trends in our study are consistent with domestic and foreign findings. Conversely, the HHcy prevalence rates in each age group of our study population were higher than those of a similar study population [20,29]. Our study showed that the prevalence of HHcy was higher in males than in females, and this difference in the prevalence of HHcy between males and females may be associated with higher creatinine concentrations in males [30,31]; additionally, the female hormone estrogen may have a role in lowering serum Hcy levels [32]. Existing data show [25] that the prevalence of hypertension in Kazakh and Uyghur males was higher than that in women. Further research is required to confirm the differences between male and female homocysteine levels observed in this study and whether the Kazakh and Uyghur gender differences in the prevalence of hypertension are part of the reason.

HHcy occurrence involves a variety of factors [5], including genetic factors, nutritional factors (vitamin B deficiency and folic acid deficiency), and lifestyle behaviors (smoking); of these factors, genetic factors and vitamin B and folic acid deficiency are the most important. Methylenetetrahydrofolate reductase (MTHFR) is a key enzyme affecting the metabolism of Hcy [33], and MTHFR activity will result in reduced Hcy metabolism, thereby causing HHcy [20]. Ilhan reported that the *MTHFR* TT genotype was associated with higher homocysteine levels in CAD patients [34]. The plasma total Hcy (tHcy) level was notably increased in acute coronary syndrome patients, and the *MTHFR* C677T TT mutation may play an important role [33]. Crider *et al*. described that *MTHFR* 677 genotypes are associated with plasma homocysteine concentrations and that the *MTHFR* 677 TT genotype is linked to higher plasma homocysteine concentrations than the CT or CC genotype [35]. In addition, the MTHFR A1298C mutant can inhibit enzyme activity, and the frequency of the 1298 C allele was significantly higher in the Kazak population than that reported in the Han population [36], which may be why Hcy levels in the former were higher than those in the latter. Vegetables rich in folic acid and other related vitamins can provide the necessary methyl group for Hcy metabolism [37]; thus, consuming small amounts of these vegetables can increase Hcy levels [38]. Chen [39] found that the folic acid levels of the Kazakh population were lower than those of the Han population, which may be another reason why the HHcy prevalence was higher in the Kazakh than in the Han. This study found that the prevalence of HHcy in Kazakh and Uyghur inhabitants was essentially the same, though the rate was significantly higher than the general prevalence of hypertension in these populations. Future studies should investigate the reasons for this difference and determine whether it is a result of differing gene expression in these populations.

Patients with H-type hypertension have a more than 12-fold greater risk of suffering from a stroke than hypertensive patients [40]. Indeed, a close relationship between Hcy and stroke has been reported in the literature, and the following five points could explain the mechanism: (1) vascular endothelial cell damage, (2) effects on vascular smooth muscle cells, (3) effects on the coagulation system, (4) altered lipid metabolism and (5) protein of Hcy. A low prevalence of H-type hypertension was previously reported in China. However, in this study of Kazakh and Uyghur populations, the prevalence of H-type hypertension was 35.1% and 30.6%, respectively, which is much higher than that estimated by Zhu [41], who reported a prevalence of 4.1% in the Han population. The prevalence of hypertension patients with H-type hypertension was 87.6 and 88.0%, respectively, which was also higher than that suggested by Hu *et al.* [8], who reported a prevalence of 75.0%. With regard to the Kazakh and Uyghur H-type hypertensive population, more effective interventions and treatments will further improve treatments and control the rates of hypertension, cardiovascular disease and cerebrovascular disease, particularly through stroke prevention.

This survey sample size is large, covers more rural minority populations, and accurately reflects the overall epidemiologic situation in rural areas. However, this study did not investigate genetic factors and environmental factors. Thus, future studies collecting genetic, dietary, environmental and nutrition information on HHcy populations will provide explanations for the high prevalence of HHcy in these two ethnic groups.
